# Optimising the release rate of naproxen liqui-pellet: a new technology for emerging novel oral dosage form

**DOI:** 10.1007/s13346-019-00659-6

**Published:** 2019-07-08

**Authors:** Matthew Lam, Taravat Ghafourian, Ali Nokhodchi

**Affiliations:** 1 Pharmaceutics Research Laboratory, School of Life Sciences, University of Sussex, Brighton, BN1 9QJ UK; 2 JMS Building, Biochemistry and Pharmacy Department, School of Life Sciences, University of Sussex, Brighton, BN1 9QG UK

**Keywords:** Liquisolid, Liquisolid pellet, Liqui-pellet, Dissolution enhancement, Extrusion-spheronisation, Liquid vehicle, Solid-state analysis

## Abstract

Liqui-pellet is a new dosage form stemming from pelletisation technology and concept from liquisolid technology. In spite of liqui-pellet overcoming a major hurdle in liquisolid technology through achieving excellent flow property with high liquid load factor, the formulation requires to be optimised in order to improve drug release rate. Liqui-pellets of naproxen containing Tween 80, Primojel, Avicel and Aerosil were extruded and spheronised. Flowability test confirmed that all liqui-pellet formulations have excellent-good flow property (Carr’s index between 3.9–11.17%), including liqui-pellets with a high liquid load factor of 1.52, where 38% of the total mass is co-solvent. This shows a relatively high liquid load factor can be achieved in liqui-pellet without compromising the flowability, which is one of the key novelty of this work. It was found that the improved drug release rate was due to the remarkably improved disintegration of the supposedly non-disintegrating microcrystalline-based pellet; the optimised liqui-pellet seems to explode into fragments in the dissolution medium. At pH 1.2, the optimised formulation had ~ 10% more drug release than non-optimised formulation after 2 h, and at pH 7.4, the drug release of the optimised pellet was nearing 100% at ~ 15 min, whereas the none-optimised pellet only achieved ~ 79% drug release after 2 h. DSC and XRPD indicated an increase in the dissolution rate could be due to molecularly dispersion of naproxen in the pellets. Overall results showed that liqui-pellet exhibited an enhanced drug release and the capacity for high liquid load factor whilst maintaining excellent flowability, rendering it a potentially commercially feasible drug delivery system.

## Introduction

Liqui-pellet is considered the novel emerging oral dosage form. It stems from liquisolid technology; however, liqui-pellet comes under liqui-mass system, which is different from liquisolid system. This liqui-mass system is combined with pelletisation technology, producing liqui-pellet [1]. In recent years, the advancement on the high-throughput screening has indicated an increased number of drug candidates to be either poorly water soluble or insoluble [2]. Since water-insoluble drugs have poor dissolution rate, the pharmaceutical industry has been challenged to overcome this in order to improve bioavailability [3]. In fact, approximately 40% of drugs in the market are considered poorly soluble in gastrointestinal fluids, and around 90% of drugs in development are identified as poorly soluble; both of which is based on biopharmaceutical classification system (BCS) [4].

**Fig. 1:**
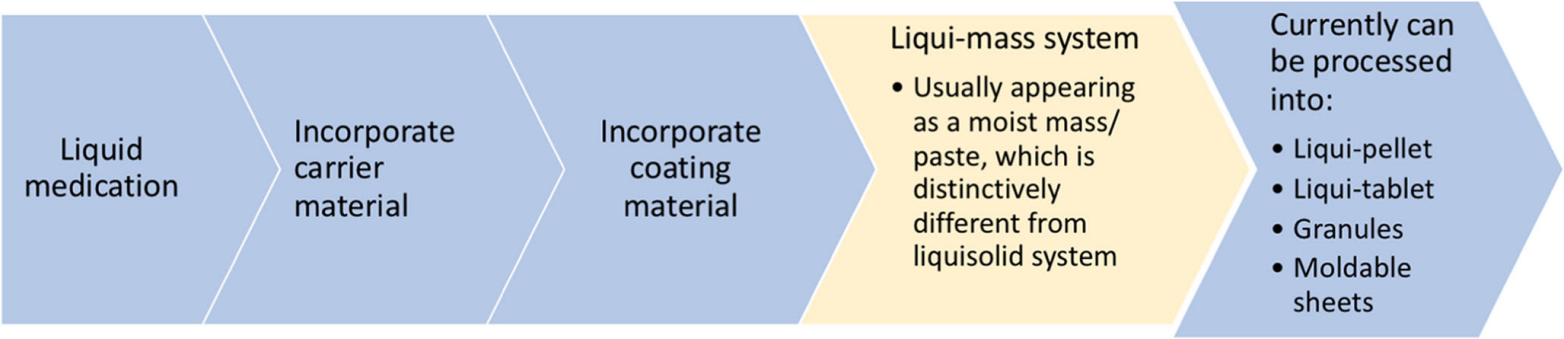
Diagram summarising the novel liqui-mass system which is used to make liqui-pellet

Liqui-mass system (Fig. 1) is similar to liquisolid system in that it comprises a solubilised active pharmaceutical ingredient (API) in an appropriate co-solvent, which form the liquid medication. This liquid medication is absorbed and adsorbed into a carrier, which is usually microcrystalline cellulose. The admixture is then coated with a coating material that is usually nanosized silicon dioxide [3, 5]. Other excipients, namely superdisintegrant, are usually added to improve dissolution rate. The major difference in liqui-mass system and liquisolid system is that the admixture of API and excipients is not necessarily a free-flowing powder in liqui-mass system, but instead can be a wet mass/paste. This is different from liquisolid system in which it is made clear that the admixture as a free-flowing powder. It should be noted that liqui-pellet is different from liquisolid pellet in that liqui-pellet is under liqui-mass system. The name liqui-pellet also aid the distinction from liquisolid pellet in that liqui-pellet is capable of high liquid load factor.

It is noteworthy to point out that the technology involving liqui-mass system is highly versatile. It can be seen in Fig. 1 that liqui-mass system encompasses wet mass/paste and but less frequently free-flowing powder. In concept, the technology concerning liqui-mass system can produce free granule, moldable sheets, liqui-pellet, liqui-tablet and more, which will be revealed in future studies. There is also a considerable flexibility for modifications of the formulation, particularly the coating of liqui-pellet. Such versatility makes this new liqui-mass system interesting and exciting to explore. In addition, this technology has major advantages such as being cost-effective, mainly uses green technology, simplistic approach, easy to scale up platform technology for commercial manufacturing, does not require organic volatile solvent, no need for an advance technique or machinery and excipient are common and easily obtainable [6]. Such advantages may not be present in other various technologies confronting the same issue of improving the bioavailability of poorly water-soluble drugs. Other technologies include conversion of crystalline drug into its amorphous state [7], solid dispersion [8]; micronisation [9–12], nanosuspension [13, 14], co-grinding [15–17], self-emulsifying drug delivery system [18, 19] and inclusion of drug solution in soft gelatin capsule [20]. It is claimed that the liqui-pellet is highly commercially feasible and has the potential to play a major role in the future oral dosage form [1].

The key purpose of liqui-pellet is to take the key advantages of liquisolid formulation into a commercially feasible dosage form. This is done by confronting the major drawbacks in liquisolid technology, such as poor flow property, poor compactibility and inability to produce high-dose drug without being too bulky and heavy for real-life use [3, 5]. In the authors’ previous studies, it is shown that liqui-pellet can achieve excellent flow property whilst having a high liquid load factor of [1]. Liqui-pellet not only contains the advantages of liquisolid technology, but it also has the inherent advantages of being in a pellet form. Such advantages include good flow property [21], potential to combine incompatible drugs or drugs with different release profiles in same dose unit [22], reduced risk of side effects due to dose dumping and the flexibility for modification via coating technology.

The aim of the present study is to optimise liqui-pellet formulation in order to improve the drug release rate of naproxen, which is a nonsteroidal anti-inflammatory drug (NSAID), belonging to BSC class II. The chosen liquid vehicle used in the investigation is Tween 80, as it was considered the most suitable liquid vehicle from previous studies [1]. Tween 80 solubilises the API as well as acting as a surface-active agent which reduces interfacial tension and improve water penetrating into the dosage form.

## Materials and methods

### Materials

Naproxen was obtained from Tokyo Chemical Industry Co (Japan). Other excipients used to prepare the liqui-pellet included microcrystalline cellulose (Avicel PH-101), (FMC corp., UK), hydrophilic fumed silica with specific surface area of 300 m^2^/g (Aerosil 300) (Evonik Industries AG, Hanau, Germany), sodium starch glycolate Type A (Primojel) (DFE Pharma, Goch, Germany), croscarmellose (Primellose) (DFE Pharma., Goch, Germany), 2-propanol (VWR Chemicals, Fontenay Sous Bois France), polysorbate 80 (Tween 80) and PEG with molecular weight of 1500 (Acros Organics, UK). All other reagents and solvent were of analytical grades.

### Preparation of naproxen liqui-pellet

The pestle and mortar method was applied to mix naproxen in Tween 80 to form the liquid medication. Avicel PH-101 (carrier) and Aerosil 300 (coating materials) were used in all the formulation with a ratio of carrier to coating material of 20. The liquid medication was then incorporated into Avicel before transferring into a mixer (Caleva Multitab, Caleva Process Solutions Ltd., UK), where the sample was mixed for 10 min at a constant rate of 125 rpm with deionised water added bit by bit to achieve reasonable plastic property for extrusion (Caleva Multitab, Caleva Process Solutions Ltd., UK). Aerosil 300 was then added into the admixture and further mixed for 10 min before extrusion. Once the sample was extruded, the extrudate was spheronised at an almost constant rotation at 4000 rpm (decrease to 3500 rpm if agglomeration seemed likely or increase to 4500 rpm to increase pellet sphericity). The spheronisation time varied depending on the extrudate plastic property to avoid agglomeration. Pellets were then placed in an oven under a constant temperature of 50 °C overnight to remove water content.

For clarity, it should be mentioned that the liqui-pellet formulations were categorised into two sections (Table 1). In the first section between LP-1 to LP-6, the main focus was to look into the effect of varying concentration of a Primojel (superdisintegrant) with and without the presence of Tween 80. In the second section of the formulations between LP-7 and LP-11, the focus was to modify the formulations in an attempt to improve drug release rate.

**Table 1:** Key formulation characteristics of the investigate liqui-pellet capsule

Formulation	Liquid vehicle	Amount of liquid vehicle (%*w*/*w*)	Liquid load factor	Primellose (%*w*/*w*)	Pre-extrusion liquid added	Amount of pre-extrusion liquid during extrusion-spheronisation (ml) per 20 g of admixture of API and excipients	Mass of carrier (mg)	Mass of coating material (mg)	Total weight of 25 mg naproxen liqui-pellet (mg)
Physical mixture pellet					Water	33.10	68.97	3.13	90.63
LP-1	Tween 80	28.23	1	5	Water	8.61	62.53	3.13	132.95
LP-2	Tween 80	27.12	1	10	Water	8.75	62.57	3.13	138.24
LP-3	Tween 80	26.04	1	15	Water	8.88	62.42	3.12	144.03
LP-4	None			5	Water	22.67	62.41	3.12	95.42
LP-5	None			10	Water	22.67	62.57	3.13	100.81
LP-6	None			15	Water	32	62.43	3.12	106.53
LP-7	Tween 80	28.23	1	5	Water	8.61	62.53	3.13	132.95
LP-8	Tween 80	38.34	1.52	5	Water	1.89	50.07	2.50	132.66
LP-9	Tween 80	28.23	1	5	Water	8.61	62.53	3.13	132.95
LP-10	Tween 80	28.23	1	5	^*^PEG and water	9.57	62.53	3.13	132.95
LP-11	Tween 80	28.23	1	5	**2-propanol and water	9.57	62.53	3.13	132.95

Note all formulation contain 25 mg of naproxen and the carrier to coating material is at a ratio of 20:1

*0.5 g of PEG was dissolved in 10 ml of water; **2-propanol and water was mixed in equal volume

### Evaluation of naproxen liqui-pellet

#### Flowability test on liqui-pellet

Three techniques were used to evaluate the flow properties of the liqui-pellet formulations, namely flow rate in grams per second (Flowability tester, Copley Scientific, UK), angle of repose (Flowability tester, Copley Scientific, UK and Digimatic height gauge, Mitutoyo, Japan) and Carr’s compressibility index using the SVM tapped density tester (D-63150, Erweka, Germany). Flow rates were measured by recording the weight (g) and time (s) of pellets flowing through a 10-mm-diameter orifice after applying the shutter. Note that shutter was applied before the funnel holding the pellet became empty. To determine the angle of repose, the pellets were placed in a funnel with 10-mm-diameter orifice and let the pellets flow onto a 100-mm-diameter circular test platform. The digimatic height gauge and micrometre were used to measure the height and diameter of the heap of the sample, so that the angle of repose could be determined. Carr’s compressibility index (CI%) was calculated using CI equation. Tapped density was measured using the tapped density tester, which was set to tap 100 times. All measurements were done in triplicates.

#### Friability test on of liqui-pellet

The friability test was adapted using a similar method as in Hu studies [23]. Two best-optimised formulations were tested. Pellets (3 g) and glass beads (3 g) were placed in Erweka friabilator (D-63150, Erweka, Germany) and sealed to prevent pellets from leaving the container. The friabilator was then rotated under the constant speed of 25 rpm for 4 min. Weight of the pellets before and after the friability test was recorded and used to calculate the percentage of weight loss.

#### Particle size analysis via sieve method

The particle size distribution was determined using sieve method (Test sieve, Retsch, Germany). Only the physical mixture pellet and two chosen optimised formulations were tested. Pellets (5 g) were sieved under vibration using the mechanical shaker (AS 200, Retsch, Germany) for 1 min with an amplitude of 50, then a further 9 min with an amplitude of 40. The size of sieves used were 2000-, 1000-, 850-, 500- and 250-μm sieves. The pellets yield was determined based on the pellet fraction between 250 and 2000 μm and presented as the percentage of total pellet weight.

#### Stereoscopic analysis

Mean Feret’s diameter along with mean roundness and mean elongation ratio was determined using an optical microscope (Nikon Labophot, Nikon, Japan) connected to a camera (Panasonic camera WVCL310, Panasonic, Japan). The image of a pellet was captured and processed using particle size analysis software V1999 (designed in-house at King’s College London). This was repeated so that 100 pellets were analysed per formulation in order to obtain Feret’s diameter and also shape factors such as roundness and elongation ratio. Roundness and elongation ratio were calculated using Eqs. 1 and 2 respectively [24]. Only physical mixture pellet and two best-optimised formulations underwent stereoscopic analysis.

1 $$ \mathrm{Roundness}={\left(\mathrm{perimeter}\right)}^2/\left(4\pi A\right) $$

2 $$ \mathrm{Elongation}\ \mathrm{ratio}=\mathrm{Maximum}\ \mathrm{Feret}\ \mathrm{diameter}/\mathrm{Minimum}\ \mathrm{Feret}\ \mathrm{diameter} $$

#### Scanning electron microscope analysis

Surface morphology studies of the liqui-pellet from each formulation were carried out using a scanning electron microscope (Jeol JMS 820, Freising, Germany). The samples were placed on a double-sided carbon tape and sputter-coated with gold using a sputter coater (Edwards S-150 sputter coater, Edwards High Vacuum Co. International, USA) with gold target and Argon gas under 5 kV for 5 min. After the samples were coated, it was ready to be placed into the scanning electron microscope (SEM) where surface structure was then observed and recorded at magnifications of × 80, × 200 and × 800, using the SEM which was operating at 3 kV.

#### In vitro drug dissolution test

The USP dissolution paddle method (708-DS Dissolution Apparatus & Cary 60 UV-Vis, Agilent Technologies, USA) was carried out for all formulations. All dissolution tests of the pellets in capsule were under the constant condition of 900 ml dissolution medium, paddle rotating at 50 rpm and temperature of 37.3 ± 0.5 °C.

Dissolution media that were used were either HCl buffer solution of pH 1.2 or phosphate buffer solution of pH 7.4 to simulate gastric fluid or intestinal fluid respectively without enzymes. The absorbance of the samples was taken at time intervals of 5 min for 1 h, and then time interval of 10 min for another hour. The authors were aware that at pH 1.2, the sink condition was not maintained; however, this pH was only used for comparison of various formulations.

All formulations contained 25 mg of naproxen and this amount of naproxen was chosen due to its poor solubility profile at pH 1.2. This weakly acidic drug would need to be able to dissolve completely at pH 1.2 in order for the dissolution test to be considered reliable. This value was also taken on the basis of its solubility at 35 °C and pH 1.2 which was 27 mg/l [25]. Although it does not follow the sink conditions, it can be used for comparison purpose for dissolution profiles of different formulations. Thus, 25 mg of naproxen seemed reasonable in this test. At pH 7.4, naproxen was extremely soluble with a solubility of ~ 3347 mg/l; hence, using 25 mg of the drug was not a problem.

### Solid-state analysis

#### Differential scanning calorimetry studies

Differential scanning calorimetry (DSC) (DCS 4000, Perkin Elmer, USA) was performed on the chosen liqui-pellet formulations with the fastest dissolution rate, including its excipients and pure naproxen in order to indirectly assess the solid state of naproxen in the formulations using data from thermograms. Samples weighing between 3 and 6 mg were sealed in aluminium pan and placed in the DSC machine with a scanning rate of 10 °C/min (from 25 to 200 °C) under nitrogen atmosphere.

#### X-ray powder diffraction studies

X-ray powder diffraction (XRPD) was performed using X-ray diffractometer (D5000, Siemens, Germany) on naproxen, excipients and selected optimised formulations in order to characterise the solid state of the materials used. Samples were scanned over a range of 2*θ* at voltage of 40 kV and current of 30 mA, with scanning angle ranged from 5° to 40° and scan rate of 0.2°/s.

The percent relative crystallinity was calculated using integrated peak method (Eq. 3) and peak height method (Eq. 4) [26]. Trapezoid method was applied to calculate the area under the curve for integrated peak method.

3 $$ \%\mathrm{XRD}\ \mathrm{relative}\ \mathrm{crystallinity}=\left({A}_{\mathrm{s}}/{A}_{\mathrm{r}}\right)\times 100 $$

4 $$ \%\mathrm{XRD}\ \mathrm{relative}\ \mathrm{crystallinity}=\left({H}_{\mathrm{s}}/{H}_{\mathrm{r}}\right)\times 100 $$

#### Statistical and mathematical analysis

Mean cumulative % drug release after 2 h from the dissolution test was statistically analysed by one-way analysis of variance (ANOVA). Results were quoted as significant where *p* < 0.05.

Difference factor (*f*_1_), Eq. 5, and similarity factor (*f*_2_), Eq. 6 [27] were used to determine the differences/similarities in terms of dissolution profile among the formulations. Such methods have been recommended by the US FDA (Food and drug administration) [28] and implemented by the FDA in various guidance documents [29, 30]. When *f*_1_ value is between 0 and 15 and *f*_2_ value is between 50 and 100, this indicates equivalence of the two dissolution profiles [31]. Details of the equations can be found in various literature [28, 32–34]. In brief, *n* represents the number of dissolution sample times and *R*_*t*_ and *T*_*t*_ represent the mean % of drug dissolved at each time point (*t*).

5 $$ {f}_1=\left\{\left[{\sum}_{t={1}^n}|{R}_t-{T}_t|\right]/\left[{\sum}_{t={1}^n}{R}_t\right]\right\}\cdotp 100 $$

6 $$ {f}_2=50\cdotp \log \left\{{\left[1+\left(1/n\right){\sum}_{t={1}^n}{\left({R}_t-{T}_t\right)}^2\right]}^{-0.5}\cdotp 100\right\} $$

## Results and discussion

### Evaluation of naproxen liqui-pellet

#### Liqui-pellet flow property

Results from the flowability tests are shown in Table 2. It is clear that all formulations have excellent or in the borderline between excellent to good flow property. Thus, it is claimed that liqui-pellet is a promising dosage form, which resolves poor flowability issue in liquisolid technology and yet maintains the inherent advantages stemming from liquisolid concept. Pezzini et al. have applied liquisolid system to pellet, but in the current study, liqui-mass system was used instead [35]. Both liqui-pellet and liquisolid pellet do indeed contain inherent advantage from liquisolid and pelletisation technologies. However, they are distinctively different in that they both use different systems (i.e. liqui-mass system versus liquisolid system). The potential of Liqui-pellet is that liquid load factor is considerably higher than what liquisolid pellet can ever achieve.

**Table 2:** Flow rate (g/s), angle of repose and Carr’s compressible index (CI%) of all liqui-pellet formulation (*n* = 3)

Formulation^a^	Flow rate (g/s) ± SD^b^	Angle of repose ± SD^b^	CI% ± SD^b^	Inference according to angle of repose	Inference according to CI%
Physical mixture pellet	8.02 ± 0.24	27.95 ± 0.14	9.08 ± 0.87	Excellent flow property	Excellent flow property
LP-1	7.60 ± 0.10	26.98 ± 0.74	5.25 ± 0.86	Excellent flow property	Excellent flow property
LP-2	7.61 ± 0.12	27.75 ± 0.31	8.13 ± 1.65	Excellent flow property	Excellent flow property
LP-3	7.42 ± 0.22	28.68 ± 0.53	6.07 ± 1.44	Excellent flow property	Excellent flow property
LP-4	10.68 ± 0.06	23.81 ± 0.40	9.95 ± 0.08	Excellent flow property	Excellent flow property
LP-5	8.59 ± 0.08	28.20 ± 0.16	11.17 ± 0.85	Excellent flow property	Good flow property
LP-6	6.96 ± 0.28	29.21 ± 0.26	10.37 ± 0.79	Excellent flow property	Excellent-good flow property
LP-7	7.13 ± 0.07	28.68 ± 0.22	7.24 ± 2.33	Excellent flow property	Excellent flow property
LP-8	5.82 ± 0.09	30.51 ± 0.38	3.90 ± 2.30	Excellent-good flow property	Excellent flow property
LP-9	7.35 ± 0.05	28.57 ± 0.50	7.63 ± 1.42	Excellent flow property	Excellent flow property
LP-10	6.47 ± 0.19	30.13 ± 0.19	9.24 ± 0.73	Excellent-good flow property	Excellent flow property
LP-11	6.03 ± 0.25	30.47 ± 0.51	7.76 ± 0.76	Excellent-good flow property	Excellent flow property

^a^For the composition of each formula refer to Table 1

^b^*SD*, standard deviation from the mean

The previous studies by the authors demonstrated that liqui-pellets achieved a high liquid load factor of 1, whilst maintaining excellent flow property [1]. In fact, before the development of liqui-pellet, it has been proven very difficult to achieve such mentioned results, due to the cohesive nature of liquid powder admixture, which is shown in various studies [36–38]. Tiong et al. formulated naproxen liquisolid powder with *L*_f_ of 0.9 but the flowability was poor (Carr’s index of 31.58) [36]. In studies by Javadzadeh et al., an additive such as PEG 3500 was used to increase the *L*_f_ [37]. They observed an increase of carbamazepine *L*_f_ from 0.25 to 0.6 [37]; however, it is clear that liqui-pellet *L*_f_ is much more superior and does not need polymeric additive. Hentzschel et al. replaced a commonly used carrier (Avicel) and coating material (Aerosil) with neusilin, a material with a much larger specific surface area (SSA) [38]. This enabled an increase of *L*_f_ by a factor of ~ 7; nonetheless, it is still limited by its flow property; their formulations’ flow rate are below 1 g/s [38].

What is exciting and promising in liqui-pellet is that it can be further optimised so that the *L*_f_ is further increased. It can be seen in Table 1 that LP-8 has *L*_f_ as high as 1.52, where 38% of the pellet total mass is co-solvent, and yet excellent-good flow property is achieved. Such a result further supports the potential of liqui-pellet being commercially feasible. This means flow property and *L*_f_ are not a major hindrance for liqui-pellets; this emerging novel oral dosage form has the potential for smooth and cost-effective manufacturing, as well as producing high-dose liqui-pellet without being overly bulky and heavy if the formulation is optimised. Although Pezzini et al. [35] overcome the flow issue of liquisolid by converting it to pellet form, the load factor calculated was around 0.1 (they did not calculate the load factor and the authors of the current research calculated it on the basis of mass data provided in Pezzini’s article), which is much lower than the load factor obtained in the current article (*L*_f_ ≥ 1). This indicates that at the same concentration of API, the final formulation in Pezzini’s article should be very bulky. In addition to load factor, the percentage of API in Pezzini formulation was around 5% (percentage of API was calculated on the basis of mass data provided in Pezzini’s article). This is lower than the majority of liqui-pellet formulation, which is around 19% (percentage of API was calculated on the basis of mass data in Table 1).

#### Friability test

Table 3 shows the results obtained from the friability test of the physical mixture pellet and two optimised formulations (LP-8 and LP-11). In brief, they all have percentage weight loss below 1%. Since there is currently no standard for friability test on pellets, USP standard friability test for tablet is adapted, which suggests less than 1% weight loss is acceptable. Therefore, it can be concluded that all tested formulations are robust, which is ideal for commercial manufacturing in terms of quality control.

**Table 3:** Weight loss of 3 g of each formulation under rotational speed of 25 rpm for 4 min

Formulation	% weight loss
Physical mixture pellet	0.54
LP-8	0.03
LP-11	0.12

It can also be postulated that the robustness is due to microcrystalline cellulose (carrier) forming sufficient bonds within its structure when water is added, hence producing robust pellets. In addition, the Tween 80 in the liqui-pellet can increase the pellet plasticity due to plasticising effect [39], which effectively increases the pellet resistant to friability.

#### Particle size analysis via sieve method

**Fig. 2:**
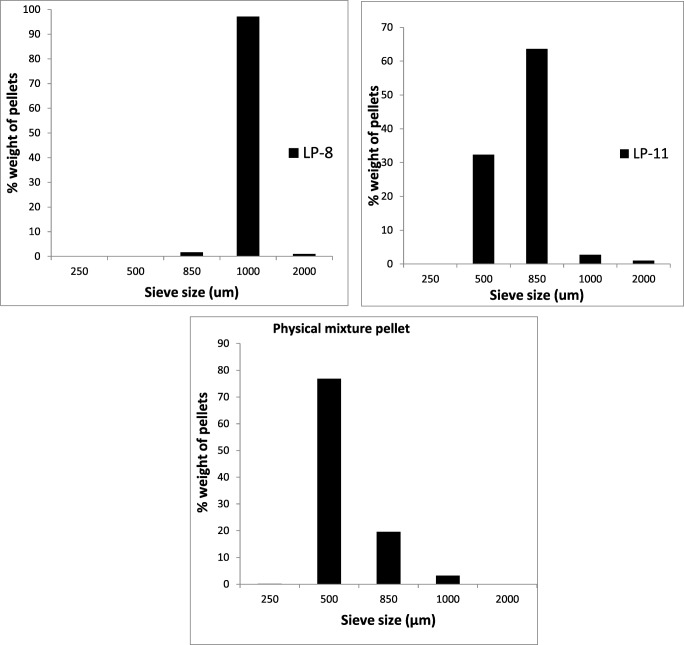
Particle size distribution of LP-8 (top), LP-11 (middle) and physical mixture pellet (bottom)

Results from particle size analysis of the optimised formulation LP-8 (Fig. 2) indicate that ~ 97% of the pellets fall in the size of 1 mm. This shows that the optimised liqui-pellet LP-8, have a good uniformity of size. This is ideal in perspective of manufacturing. There will be less likelihood of variation in volume of pellet during encapsulation, consequently maintaining uniformity of drug content during filling process.

Particle size analysis of LP-11 shows a wider size distribution with ~ 64% fall in the size of 850 μm and ~ 32% fall in the size of 1 mm. This could be due to the reduced plastic property as a result of a reduction in water content, leading to a poorer quality pellet with wider size distribution.

Nonetheless, both of the optimised formulations are almost entirely below 2-mm range which will behave similarly to liquid in the stomach and be emptied into the small intestine relatively fast [40]. This can be beneficial for weakly acidic drugs (i.e. naproxen), as they are more soluble in an alkaline environment, suggesting that bioavailability and onset of action may be improved.

It is also interesting to note that most of the physical mixture pellet falls in 500 μm, which is considered small. This supports the claim from the previous studies by the authors that co-solvent tends to increased pellet size [41].

#### Stereoscopic analysis

The Feret’s diameter (Table 4) agrees with the trend that co-solvent tends to increase liqui-pellet size. It can be seen that physical mixture and LP-11 mean Ferret diameter overestimated the liqui-pellet size. Since the pellets are not perfectly spherical, they tend to be in their most stable orientation. This means that the smallest dimension is orientated vertically; therefore, overestimation is prone to occur [42].

**Table 4:** Stereoscopic analysis showing the mean Feret’s diameter, mean roundness and mean elongation of physical mixture pellet and optimised formulation (*n* = 100)

Formulations^a^	Mean Feret’s diameter (mm)	Mean roundness ± SD^b^	Mean elongation ratio ± SD^b^
Physical mixture pellet	1.028	1.25 ± 0.12	1.41 ± 0.19
LP-8	1.431	1.28 ± 0.14	1.43 ± 0.21
LP-11	1.527	1.42 ± 0.12	1.95 ± 0.35

^a^For the composition of each formula, refer to Table 1

^b^*SD*, standard deviation from the mean

It is clear that LP-11 is the least round with mean roundness deviating from 1 considerably (1.42). Its mean elongation ratio is also large which supports the visible observation of the cylindrical liqui-pellet. Perhaps the reduced plastic property due to decreased water content leads to incomplete spheres forming. Nonetheless, LP-11 has excellent-good flowability.

#### Scanning electron microscope analysis

**Fig. 3:**
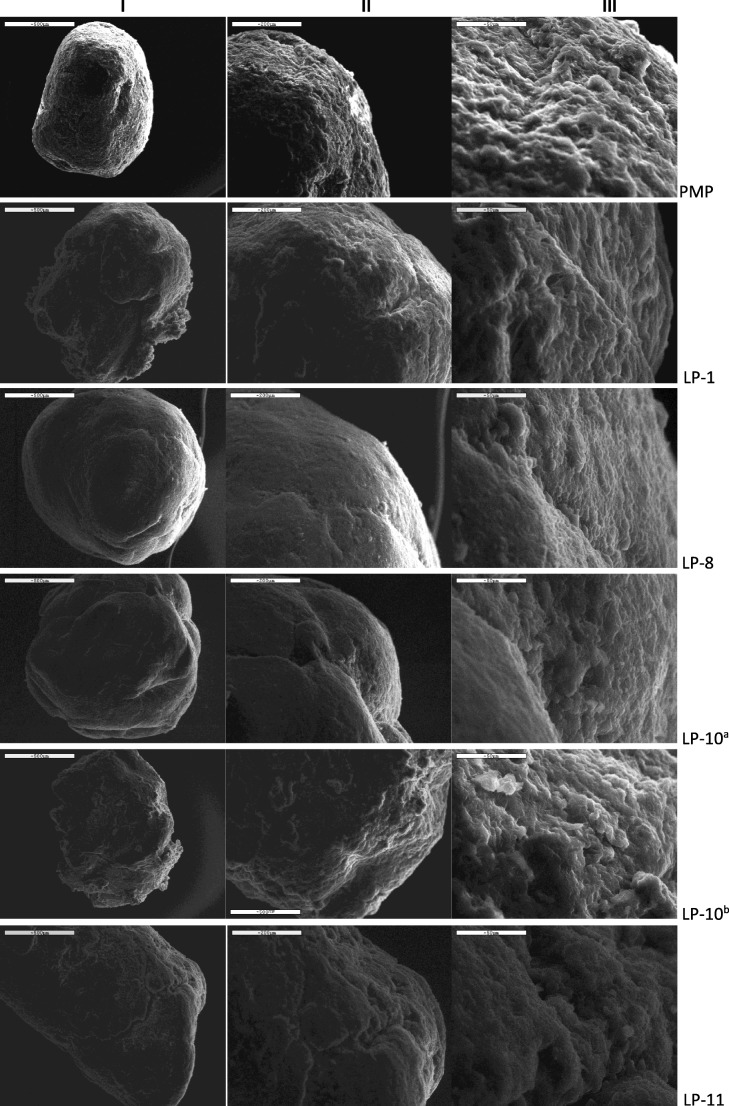
Images from SEM of physical mixture pellet, LP-7, LP-8 and LP-11; (I) × 80 magnification, (II) × 200 magnification and (III) × 800 magnification

The SEM results (Fig. 3) show physical mixture pellet (PMP) has the roughest surface structure compared to the rest of the formulations. This can be seen clearly at × 800 magnification. Although formulation LP-1, which contains 28% Tween 80, shows surface crack at × 80 magnification, its surface is less rough than the physical mixture pellet. This is more apparent at × 200 and × 800 magnification. The formulation LP-8, which contains 38% Tween 80, shows a remarkable reduction in surface roughness compared to LP-1. This indicates that surface structure becomes less rough as Tween 80 is increased.

The observation supports the claim made from the previous studies by the authors that co-solvent influences surface structure, which tends to make the surface smoother [1]. It is speculated from the previous studies that liquid vehicle may reduce the crystallinity of the pellet, resulting in smoother surface structure.

The SEM image of formulation LP-11 shows a large number of cracks in the pellet compared to LP-8. In LP-11, which also contains Tween 80 as liquid vehicle, the water content is reduced during the manufacturing of the liqui-pellet. Water and 2-propanol mixture is used instead of just water to reduce overall water content during production. With a reduced amount of water, there will be less bonding within the MCC structure; thus, the pellet quality is reduced. Nonetheless, this is advantageous as the pellet was able to disintegrate well in dissolution medium.

In formulation LP-10, PEG (molecular weight of 1500) and water mixture is used to make the liqui-pellet. It is thought that the PEG at the surface of the pellets will dissolve faster, forming pores. However, it is clear in Fig. 3 image LP-10^b^, there is no apparent porous structure but the surface did become rougher.

#### Drug release study

As mentioned in the previous studies by the authors, naproxen has poor solubility in acidic condition; however, for comparison purpose, the dissolution of liqui-pellets was carried out at pH 1.2. The authors have found that Tween 80 appears to be the most suitable co-solvent for naproxen liqui-pellet [41]; hence, Tween 80 is the chosen liquid vehicle in this study. As observed in the previous dissolution study of liqui-pellet, the lack of disintegrating properties of Avicel led to poor drug dissolution rate [1]. In an attempt to promote disintegration, a superdisintegrant (Primojel) with different concentrations (5, 10, 15% *w*/*w*) is introduced into the formulation.

**Fig. 4:**
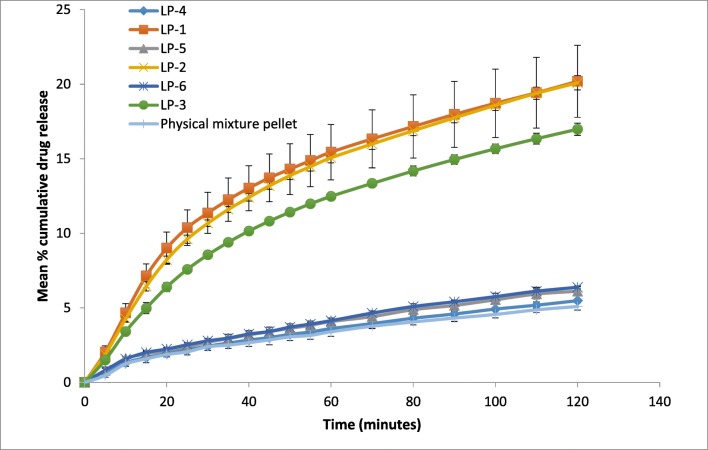
Dissolution profile of pellets in capsule for naproxen 25 mg with different concentrations of Primojel (5, 10 and 15% *w*/*w*) with and without Tween 80 (pH 1.2)

Dissolution test of the formulations containing 5%, 10% and 15% *w*/*w* of Primojel with and without liquid vehicle is shown in Fig. 4. Liqui-pellet formulations containing 5 and 10% *w*/*w* Primojel (LP-1 and LP-2 respectively) had similar dissolution profiles (*F*_1_ = 3.3 and *F*_2_ = 97.84). It can be seen that increasing Primojel concentration to 15% *w*/*w* (LP-3) slightly impedes dissolution by ~ 5% in comparison to formulations containing 5% *w*/*w* (LP-1) and 10% *w*/*w* (LP-2) Primojel. When comparing LP-1 (Primogel 5% *w*/*w*) and LP-3 (Primogel 15% *w*/*w*), *F*_1_ = 18.91 and *F*_2_ = 80.81. It can be seen the *F*_1_ value mentioned is above 15, indicating differences in the drug release profile due; however, the *F*_2_ value suggests equivalence. If *F*_1_ is favoured over *F*_2_ and assuming 15% Primojel is slowing down drug release compared to 5% Primojel, this may be due to Primojel forming gel, which can slow down drug release rate. Literature claims that the required concentration of Primojel to achieve optimum disintegration action is ~ 4% *w*/*w* [43]. Given this claim, Primojel with concentration of 5% *w*/*w* is chosen as opposed to 10% for the other formulations. Results from Fig. 4 confirm formulation without a liquid vehicle has considerably slower drug release rate compared to the one with liquid vehicle (*p* < 0.05). In fact, even with different concentration of Primojel incorporated into the formulation (LP-4, LP-5 and LP-6), the dissolution profiles are similar to that of physical mixture pellet (*p* > 0.05). This further confirms that the characteristic of enhanced drug release in liquisolid formulations can be maintained in liqui-pellet.

**Fig. 5:**
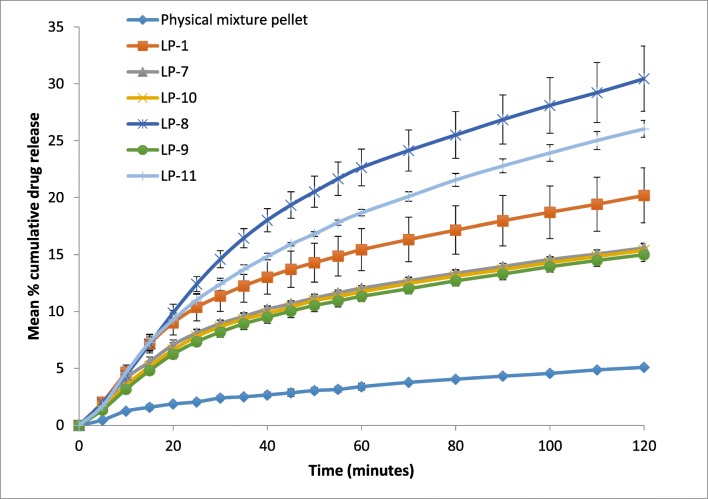
Dissolution profile of pellets in capsule for naproxen 25 mg with various modifications in attempt to improve dissolution rate (pH 1.2)

After the most suitable liquid vehicle and concentration of Primojel are chosen, various modifications are applied to the formulation to further improve the drug release rate. As seen in Fig. 5, a formulation containing an increased Tween 80 and decreased carrier and coating materials (LP-8) shows the best enhanced drug release profile.

**Fig. 6:**
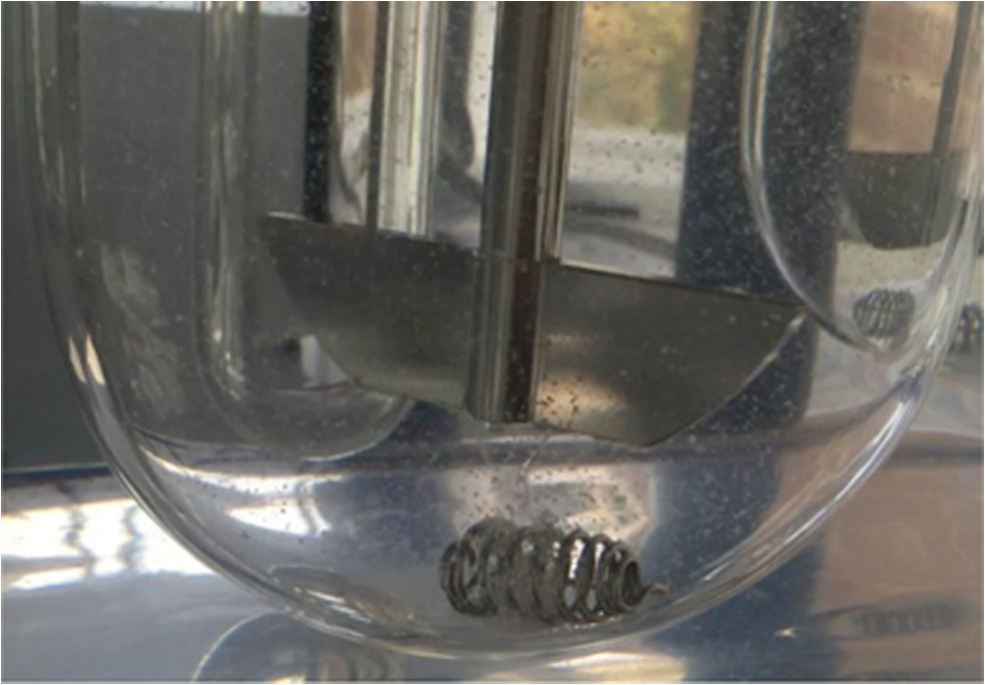
An image of LP-8 liqui-pellet disintegrate explosively in acidic dissolution medium. Note the small white specks are fragments of the liqui-pellet

When comparing LP-8 to the non-optimised naproxen liqui-pellet containing Tween 80 (LP-1), it can be seen that the drug release from LP-8 is ~ 10% higher (*p* < 0.05) and ~ 25% higher than physical mixture pellet (*p* < 0.05) after 2 h. This shows the potential of increasing drug dissolution rate of liqui-pellet when the formulation is optimised. In addition to LP-8 having the fastest drug release rate, its liquid load factor is higher than other formulations (*L*_f_ = 1.52), whilst still maintaining excellent-good flow property. With the increase in Tween 80, less water is required to achieve the appropriate level of plasticity of the extrudate for making quality pellets when spheronised. This is due to the Tween 80 plasticising effect [39]. With less water included in the formulation, it can be deduced that the amount of bonding within the microcrystalline cellulose structure is reduced. Thus, disintegration is more rapid, which is visibly observable during dissolution test (Fig. 6). In fact, the disintegration is rather fast and explosive, which is the reason for higher drug release rate. One of the limitations of microcrystalline cellulose carrier in pelletisation via extrusion-spheronisation is the difficulty of achieving enhanced drug release due to strong bonding, rendering the pellet none disintegrating [44]. In spite of this, microcrystalline cellulose is used because it is the gold standard in extrusion and spheronisation technology as it has the proper rheological properties, cohesiveness and plasticity to yield strong spherical pellets [45]. Formulation LP-11 has the second best enhanced drug release rate (~ 26% within 2 h). This can be explained in a similar manner to LP-8, where 2-propanol and water mixture is used during the liqui-pellet preparation, which effectively reduces the amount of water. Hence, bonding force within microcrystalline cellulose is reduced, leading to improvement in the propensity for disintegration.

It is found that the stage at which Primojel is added during formulation has a slight effect on how well the superdisintegrant performs. When Primojel is added into the liqui-mass system after coating material (LP-7), the drug release rate is ~ 5% lower than the same formulation where Primojel is added in the early stage along with the carrier (LP-1). The *F*_1_ = 27.81 and *F*_2_ = 74.83. *F*_1_ indicates a difference in their dissolution profile but *F*_2_ indicates equivalence. It should be noted that the range of *F*_1_ and *F*_2_ indicating difference or equivalence is only a suggestion from FDA [32] and the basis of the criteria for determining the difference and similarity between dissolution profiles are unclear. In fact, *F*_2_ is insensitive to the shape of the dissolution profile and is a sample statistic that cannot be used in formulating a statistical hypothesis for the assessment of dissolution similarity [32, 34]. This makes it impossible to evaluate false positive or false negative. With the assumption that *F*_1_ is reliable, the stages of when Primojel is added does have an influence on drug release rate. It can be seen that the superdisintegrant is added extragranularly in LP-7 and intragranularly in LP-1. Intragranular incorporation of Primojel appears to be more effective than extragranular for improving drug release. This reflects the importance of having an optimum procedure for preparing liqui-pellet. In literature, a combination of intragranular and extragranular incorporation of superdisintegrant is most effective in promoting disintegration [46–48].

In formulation LP-9, Primojel superdisintegrant is replaced by Primellose to see if the sodium starch glycolate or croscarmellose sodium (respectively) will perform better. Results from Fig. 6 shows Primojel (LP-1) have ~ 4% more drug release than Primellose (LP-9) after 2 h (*F*_1_ = 36.94 and *F*_2_ = 71.10). If *F*_1_ is taken into account, then this suggests Primojel is the better superdisintegrant of choice for naproxen liqui-pellet.

In formulation LP-10, PEG (molecular weight of 1500) and water mixture was used to make the liqui-pellet. It is thought that the PEG at the surface of the pellets will dissolve faster, forming pores which can facilitate the penetration of water into pellets; or that the liquid medication can move out easily via the pores generated as a result of the dissolution of PEG in dissolution medium. However, the results show similar drug release rate to that of LP-7; thus, no improvement in dissolution rate is observed. The SEM results (Fig. 3) show that LP-10 surface is rougher after the dissolution test, but the porous structure is not apparent. Without the porous structure, the drug release rate would not improve.

**Fig. 7:**
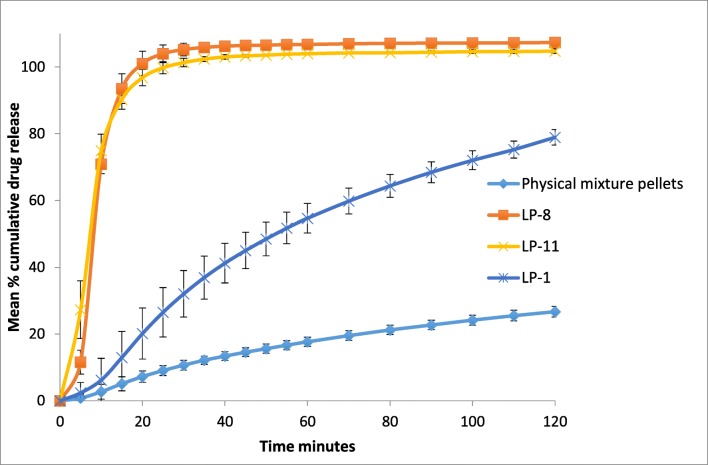
Dissolution profile of formulations containing naproxen 25 mg with the fastest dissolution rate after modifications, formulation containing Tween 80 as liquid vehicle with Primojel 5% *w*/*w*, and physical mixture pellets (pH 7.4)

USP pharmacopoeia suggests performing the dissolution test at pH 7.4 to maintain sink conditions. On the basis of this, the authors believe that those formulations which show higher dissolution at pH 1.2 should exhibit the better dissolution at higher pH; therefore, only the optimised formulation (LP-8 and LP-11) is selected for dissolution test at pH 7.4 (Fig. 7). The results show both formulations are reaching near plateau after 20 min (~ 100% drug release within 20 min). This fast dissolution profile is to be expected, as naproxen is a weakly acidic drug; hence, it will dissolve more rapidly in a basic environment. In brief, the results show naproxen liqui-pellet is capable of achieving a fast release rate even though there have been claims that microcrystalline cellulose-based pellets prepared via extrusion-spheronisation tend to prolong drug release [49]. In addition, since the pellets are small, i.e. ~ 98.8% of both LP-8 and LP-11 fall into the size of 1 mm or below; these pellets will undergo gastric emptying relatively fast, similar to liquid [40]. It will be exposed to basic environment relatively quick; thus, drug dissolution should occur faster and potentially improve the drug bioavailability. Also, since the pellets are small, it will be well distributed along the gastrointestinal tract, which could further improve bioavailability [40].

According to results shown in Fig. 6, it seems that water content during the preparation of the liqui-pellet plays a major role in disintegration and drug release rate. It is clear that reducing the water content causes a significant improvement in drug release, most likely due to reduced bonding force within the microcrystalline cellulose structure, which improves disintegration. Furthermore, Avicel has disintegrant properties [43], which are displayed with reduced water content formulations. The drug release rate of the reduced water formulations (LP-8 and LP-11) nearly reached towards the plateau after 20 min, whereas a formulation without reduced water content (LP-1) only has ~ 79% drug release after 2 h.

When comparing the results from Figs. 5 and 7 to Tiong et al. studies [36] on naproxen liquisolid compact, liquisolid tablets showed faster drug release rate at pH 1.2; however, at pH 7.4, the dissolution rate for the optimised formulations are similar or slightly better than Tiong et al. With excellent-good flow property being achieved in liqui-pellet and the intrinsic advantages of liqui-pellet, including possible room for further modifications, the novel liqui-pellet seems like a promising approach in tackling bioavailability issue of poorly water-soluble drugs in a commercially feasible and cost-effective way.

#### DSC studies

**Fig. 8:**
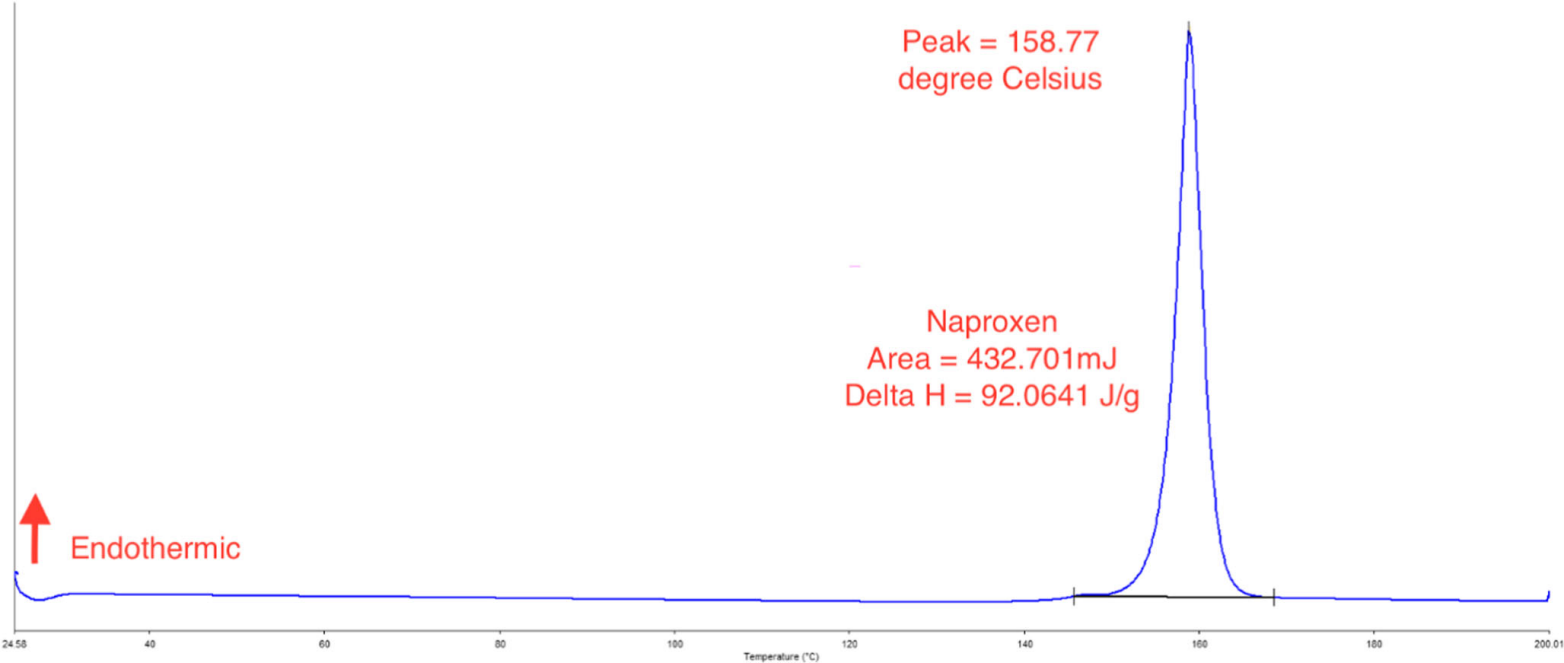
DSC thermogram of naproxen

**Fig. 9:**
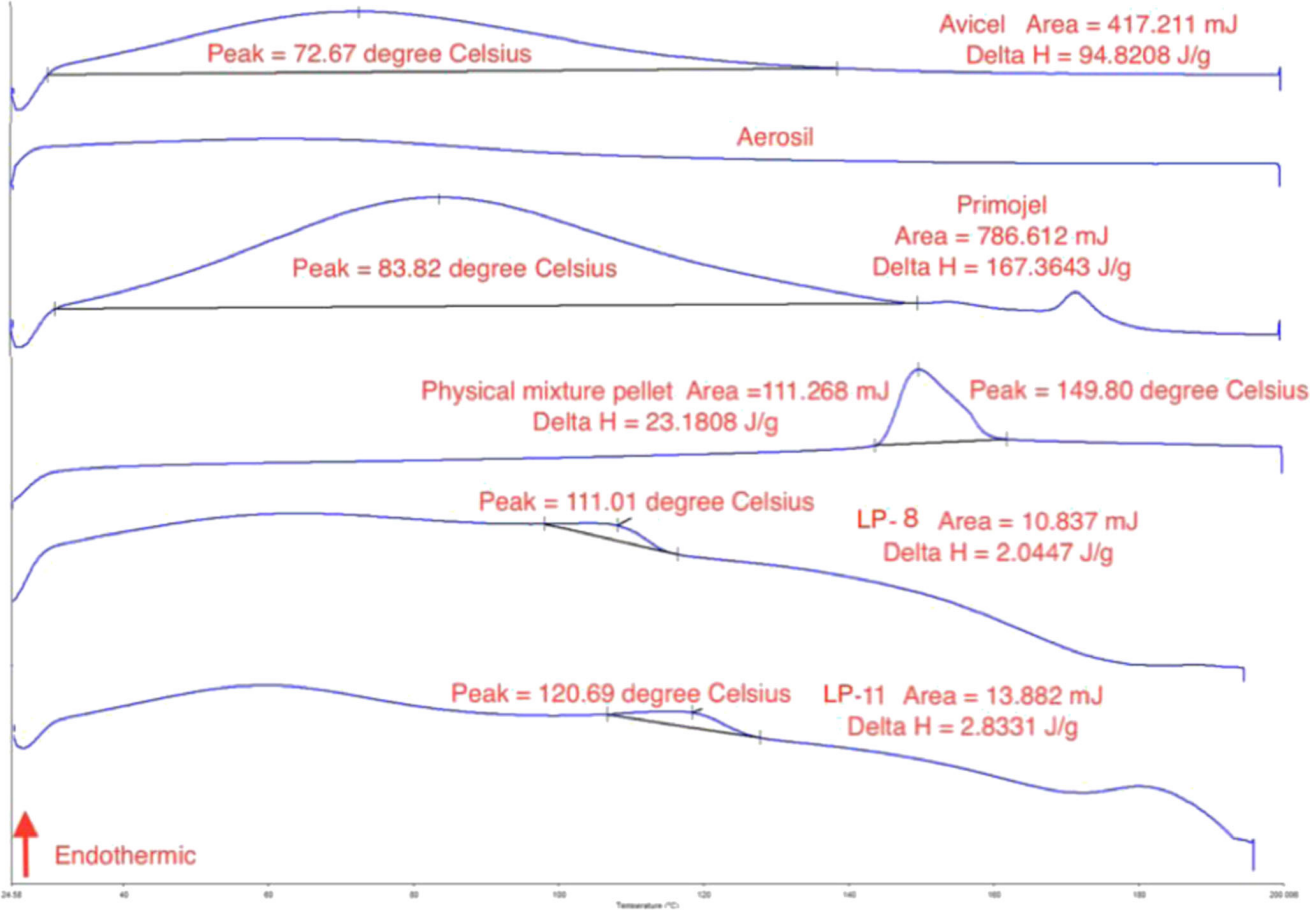
DSC thermograms of Avicel, Aerosil, Primojel physical mixture pellet, LP-8 and LP-11. Note the scales of Avicel, Aerosil and Primojel are the same but different from physical mixture, LP-8 and LP-11

The DSC traces of naproxen, Avicel, Aerosil, Primojel, physical mixture pellets and some optimised liqui-pellet formulations are shown in Figs. 8 and 9. The naproxen trace shows a sharp endothermic peak (*T*_m_ = 158.77 °C and Δ*H* = 92.06 J/g) indicating its crystalline state. Avicel (*T*_m_ = 72.67 °C and Δ*H* = 94.82 J/g) and Primojel (*T*_m_ = 83.82 °C and Δ*H* = 167.36 J/g) thermograms displayed broad peak. These peaks could be due to water within Avicel and Primojel evaporating, as they are hygroscopic materials. Tiong et al. also observed the evaporation of water from Avicel [36]. As for Aerosil, there was no definitive peak.

When comparing naproxen and physical mixture pellet thermograms as shown in Figs. 8 and 9, it can be seen that there is a small shift of peak from 158.77 to 149.80 °C respectively. This could be due to Avicel influencing the overall peak of naproxen in the physical mixture pellet. Nonetheless, the crystalline state of naproxen is still present. However, when looking at the DSC traces of optimised formulations (liqui-pellets), LP-8 (*T*_m_ = 111.01 °C and Δ*H* = 2.04 J/g) and LP-11 (*T*_m_ = 120.69 °C and Δ*H* = 2.83 J/g), the naproxen peak was absent and the *T*_m_ lowered, indicating that they were less crystalline and possibly more amorphous, hence the improvement in dissolution.

#### XRPD studies

**Fig. 10:**
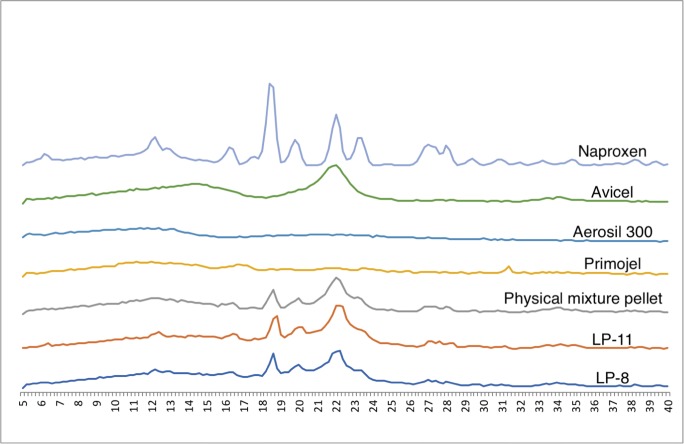
Diffractograms of naproxen, Avicel, Aerosil, Primojel, physical mixture pellet, LP-8 and LP-11

Figure 10 shows major peaks of naproxen at 2*θ* values of 12.2, 16.2, 18.4, 19.6, 22.2, 23.2, 26.8 and 27.8° which are also reported by Maghsoodi et al. [50], with the exception of a sharp peak at ~ 7° being present and peak at 26.8° being absent in Maghsoodi studies. Another research carried out by Mello and Ricci-Junior [51] showed some variation in naproxen peaks between different studies. This could be due to different scan rate settings or the actual state or form of the drug used in various studies. Nonetheless, the general peaks of naproxen are present.

The XRPD diffractogram of physical mixture pellet and formulation LP-8 and LP-11 has no peak other than that of naproxen and Avicel, which indicates no interaction between the excipients and the drug (Fig. 10). As in LP formulations, naproxen is in a molecularly dispersed state; therefore, it is expected to have more halo XRPD compared to the physical mixtures. Figure 9 does not show a big difference in XRPD between the physical mixtures and LP formulations. This could be due to the presence of a high concentration of amorphous Avicel which overshadow the overall XRPD peaks in the physical mixtures. The percentage crystallinity was calculated on the basis of Eqs. 3 and 4 showed that LP8 had the lowest crystallinity (18% and 36% using Eqs. 3 and 4 respectively) compared to physical mixtures which are 23% and 40%. The authors believed that the crystallinity of the physical mixtures should be much higher than the reported values in this study which could be due to the presence of a high concentration of Avicel in the sample as described earlier.

## Conclusion

It is confirmed that optimised liqui-pellet is capable of enhanced drug release when propensity for disintegration is improved. Although Avicel is known to be non-disintegrating, when the water content is reduced during liqui-pellet production, the pellet is capable of fast and even explosive disintegration. The major drawback of classical liquisolid formulation having poor flowability has been overcome by replacing it with the new liqui-pellet dosage form. All liqui-pellet formulation maintained excellent-good flow properties even with an extremely high liquid load factor of 1.52, where 38% of total pellet mass is co-solvent. In conclusion, it is reasonable to postulate that liqui-pellet is highly commercially feasible without having the advantages of liquisolid formulation compromised. Furthermore, there is potential for further optimisation of this novel delivery system as the parameters have yet to be optimised.
